# Impact of some social and clinical factors on the development of postpartum depression in Chinese women

**DOI:** 10.1186/s12884-020-02906-y

**Published:** 2020-04-16

**Authors:** Qing Li, Shunyu Yang, Ming Xie, Xiaoming Wu, Liping Huang, Weiqing Ruan, Yungang Liu

**Affiliations:** 1 Department of Dietetics, Nanfang Hospital, Southern Medical University, 1838 N. Guangzhou Avenue, Guangzhou, 510515 China; 2 Department of Obstetrics and Gynecology, Nanfang Hospital, Southern Medical University, 1838 N. Guangzhou Avenue, Guangzhou, 510515 China; 3 Department of Health Management, Nanfang Hospital, Southern Medical University, 1838 N. Guangzhou Avenue, Guangzhou, 510515 China; 4 School of Public Health, Southern Medical University, 1023 S. Shatai Road, Guangzhou, 510515 China

**Keywords:** Traditional Chinese culture, Postpartum depression, Delivery mode, Feeding pattern

## Abstract

**Background:**

Postpartum depression (PPD) is prevalent and may present major adverse impacts on mother and child health. According to previous studies, mostly from the western society, PPD may have complicated etiologies, such as genetic, social and psychological factors. The aim of this study was to explore the associations of some social and clinical factors, particularly those unique in Chinese, with significant PPD symptoms.

**Methods:**

A sample of 556 pregnant women in their 36th to 40th gestational week were randomly recruited in a cross-sectional study using a self-reported questionnaire, which collected maternal sociodemographic and clinical information. During their 2nd to 4th postpartum months, 522 participants responded to our screening of significant PPD symptoms, based on a score of Edinburgh Postnatal Depression Scale ≥9.

**Results:**

A total of 90 (17.3%) participants were identified with significant PPD symptoms, and the following factors were observed more frequently in women with significant PPD symptoms (PPD^+^) than with fewer symptoms (PPD^−^): intensive involvement of parents-in-law in a participant’s life (living together with her, taking care of her, or discriminating against a female baby), lack of support from husband, cesarean delivery, and breast milk insufficiency (supplemented with formula). After multiple logistic regression analysis, parents-in-law’s preference to baby boy while devaluing baby girl, dissatisfaction with husband’s support, cesarean delivery, and mixed feeding were strongly associated with significant PPD symptoms.

**Conclusion:**

The potential risk factors for significant PPD symptoms, i.e., “son preference” custom, cesarean delivery and mixed feeding, deserve confirmation in continued, especially clinical diagnosis-based longitudinal studies.

## Background

Postpartum depression (PPD) is a type of depression that affects some postnatal women, typically occurring 4 to 6 weeks after giving birth to an infant, with its symptoms including sadness, changes in sleeping and eating patterns, low energy, anxiety, and irritability [1]. The incidence of PPD in different regions/populations varies widely. Some investigations indicate that it ranges from 10 to 20%, but the values may be influenced by the assessment method applied, the timing of the investigation, and the cultural characteristics of the population [2, 3].

The development of PPD influences not only the personal lives of the involved postpartum women and their families, but also their relatives and social connections, particularly their parents and parents-in-law [4]. Women with PPD are susceptible to insufficient breastfeeding (with reduced frequency and amount), compromised care for the babies and themselves, lack of communication with social connections, bad relations with their family members, and committing suicide (in case of extremely severe episode of PPD). Maternal depression has been linked to poor infant physical growth, infant malnutrition and increased prevalence of other pediatric diseases, such as respiratory and diarrheal illnesses [5].

Multiple risk factors may be involved in the development of PPD, which include genetic predisposition, and various environmental, social, psychological, and biological factors [6, 7]. Relevant studies, mostly carried out on Caucasian women in western countries, suggest that marital conflict and violence, low income, immigrant status, personality disorders and several other factors are associated with PPD [8–10]. These factors, however, may be reflected in China in a different way, or there might be some risk factors for PPD which uniquely exist in the Chinese population, considering some traditional cultures and customs, such as parents’ and/or grandparents’ preference of a male baby and discrimination against a female baby, intensive involvement of the parents-in-law in a pregnant/postpartum woman’s life, and the resultant conflict between them due to different life styles and ways to care for the infant. Moreover, a surgical procedure performed on a woman during delivery might bring some psychological stress, in addition to physical sufferings, since in China a hospitalized patient regularly lives in a ward with one to several wardmates (depending on the area of a ward, a restroom being only set up for a large ward). However, in some western countries, such as the USA, private hospitals are dominant with single wards optional to women for delivery and hospitalization; in such circumstances their privacy can better be respected. Based on common sense surgical procedures such as cesarean section may bring more significant psychological stress to a woman giving birth in a hospital in China than in some developed countries. Therefore, the major objective of this study was to investigate various sociodemographic and clinical factors, in particular some Chinese-featured ones, in regard to their associations with the development of PPD symptoms.

## Methods

### Study design and participants

This cross-sectional study was performed at the out-patient service of the Department of Obstetrics and Gynecology, Nanfang Hospital, an affiliate to Southern Medical University, from January, 2017 to July, 2018. The inclusion criteria were: pregnant women aged between 18 and 45 years, at gestational week 36–40, being native Cantonese or otherwise having lived in Guangzhou or other areas in Guangdong Province for more than 10 years, and have completely answered the questions from an interviewer. Exclusion criteria: women with a history of major psychosis, such as schizophrenia, bipolar affective disorder, major depression, obsession, and cerebral diseases (e.g., a history of brain surgery), which are already confirmed etiological factors for PPD (this study was designed to investigate more subtle and novel factors for PPD). A total of 556 pregnant women who met the inclusive criteria were recruited for a baseline survey in which the demographical and clinical conditions of the participants were collected. During the 2nd to 4th postnatal months, a survey of the participants for PPD symptoms using the Edinburgh Postnatal Depression Scale (EPDS) was performed.

### Ethical considerations

All participants provided written informed consent, and the study was approved by the Nanfang Hospital Ethics Committee. Since no intervention measures or invasive procedures on the participants in this study were present, our institutional ethical committee suggested that no ethical approval documents were required.

### Measurements

A two-part self-reported questionnaire, one for prenatal and the other for postnatal investigation, were conducted by three trained interviewers to obtain information on maternal sociodemographic and clinical data. The prenatal questionnaire was performed by face-to-face interviews in the hospital, while the postnatal questionnaire was completed by follow-up telephone calls. In the first part, the basic sociodemographic and clinical features were collected before delivery, while in the second part the factors suspected to be of relevance to depression, were investigated after delivery. The self-administered questionnaire at the baseline survey inquired the participant’s age, bodyweight, height, education level, occupation, gestational week, obstetric history, smoking habits, family structure, family income, husband/parents-in-law’s attitude toward a female baby, and pre-pregnancy history of mild mental problems (such as anxiety and low mood). The items on the postnatal questionnaire included feeding pattern, participants’ care givers, self-evaluation on husband’s support in puerperal care, and the occurrence of venting emotions on baby (by frightening or beating baby). The pregnancy outcome (with or without delivery complications, e.g., postpartum hemorrhage, laceration of perineum, and puerperal infection), delivery mode, and infant’s gender and health status were obtained from the medical record of each participant.

In a subsequent survey, Edinburgh Postnatal Depression Scale, a 10-item self-reported scale designed to screen for symptoms of PPD [11], was employed, in which interviews were conducted during the 2nd to 4th postpartum months after delivery using a telephone call to obtain information related to PPD. Each item was scored on a four-point scale (from 0 to 3), giving a total score ranging from 0 to 30, EPDS score being increased with the symptom severity. The scale indicates the intensity of depressive symptoms occurring in the recent 7 days [11]. Although a cut-off of 9/10 has been proposed to identify PPD symptoms, a cut-off as 8/9 was used in this survey, which might be optimal for the identification of PPD symptoms among Chinese women (with a specificity of 93% and a sensitivity of 75%) [12]; thus, in this study significant PPD symptomatology (elevated PPD symptoms) was judged according to an EPDS score as ≥9.

### Statistical analysis

Descriptive data on the characteristics of pregnant women were expressed as means and standard deviations. One-way ANOVA was performed to assess the continuous variables between groups. The χ^2^ test was used to compare the observed frequencies between various groups. Multiple logistic regression analysis was used to examine the relevance of delivery mode, feeding pattern, discrimination of a female baby by husband and parents-in-law, and other factors, to the occurrence of PPD symptoms. Statistical comparisons were considered significant at *p* ≤ 0.05. All statistical analyses were performed using the software package SPSS, version 18.0.

## Results

### Baseline characteristics of participants

The basic characteristics of participants were indicated in Table 1. Of the recruited 556 participants who completed prenatal baseline questionnaire, 522 (93.8%) received and responded to postnatal follow-up phone calls for EPDS and other questions on each participant’s postnatal life. A total of 432 (82.7%) participants were identified as without (PPD^−^), and 90 (17.3%) as with, clinically significant PPD symptoms (PPD^+^), according to the criteria described above. The participants were aged from 18 to 44 years, and the pre-pregnancy BMI was 21.0 ± 3.0 (means ± S.D). Of these women, 18.1% completed only primary (6-year) education, 16.7% finished secondary (9-year) or high (12-year) school, and 65.2% finished higher education (at least 3 years after high school). 28.0% of the women were unemployed, 72.0% were employed with a permanent or transient job.

**Table 1:** Socio-demographical characteristics of the pregnant women participating in the study

Variable	Mean, S. D. / n%
Age (years)	30.6, 4.9
Pre-pregnancy BMI (kg/m^2^)	21.0, 3.0
Gestation (weeks)	38.0, 5.0
Education level ^a^, %	
Primary school	18.1
Secondary & high school	16.7
Higher education	65.2
Occupation (%)	
Employed with a permanent or transient job	72.0
Unemployed	28.0

Data are based on information collected by face-to-face interviews of a total of 556 pregnant women at their 36th ~ 40th gestational weeks

^a^ Primary, secondary and high school education covers a period of 6, 9, and 12 years, respectively; and higher education means a total of at least 15 years of education

A total of 522 participants’ postpartum experiences during their 2nd to 4th postpartum months were evaluated with EPDS, by follow-up telephone calls. Thirty-four participants could not be contacted successfully, as some did not answer the phone calls or responded to questions, whilst others’ telephone numbers became invalid.

### Exposure of some prenatal factors in women with and without significant PPD symptoms

Risk factors were assessed based on questionnaire data collected in the period between the last ultrasound examination prior to delivery, normally scheduled at the 36th week of pregnancy, and the birth of the infant(s). As indicated in Table 2, no significant differences were observed between the women in the PPD^+^ and PPD^−^ groups in the following parameters: high risk pregnancy, history of mild mental problems (demonstrated by mild or moderate anxiety or low mood, rather than major psychiatric diseases), and the size of the participant’s house. The rates of discrimination against a female baby by husband between the two groups were not statistically different. However, a statistically significant difference between the women in the PPD^+^ and PPD^−^ groups in the rates of discrimination against a female baby by parents-in-law was observed (*p* = 0.028). With regards to the family member(s) who live together with a postpartum woman (very common in China), the rate for the participants’ biological parents was higher in the PPD^−^ (29.4%) than PPD^+^ women (11.1%), while that for parents-in-law was higher in the PPD^+^ (54.4%) than PPD^−^ women (36.3%), and that for husband only seemed identical in both groups (34.3 versus 34.5%). The different types of family members living together with the participants were differently distributed in the women with and without significant PPD symptoms (*p* = 0.025).

**Table 2:** Prenatal exposure of some socio-demographical factors in women with and without significant PPD symptoms

	PPD^−^ women (n, %)		PPD^+^ women (n, %)		*χ*^2^	*p*
High risk pregnancy					8.473	0.399
Yes	212	49.1%	58	64.4%		
No	220	50.9%	32	35.6%		
Discrimination against a female baby by parents-in-law					11.145	0.028
Yes	159	36.8%	53	58.8%		
No	273	63.2%	37	41.2%		
Discrimination against a female baby by husband					6.436	0.217
Yes	85	19.7%	32	35.6%		
No	347	80.3%	58	64.4%		
Pre-pregnancy history of mild mental problems					6.921	0.257
Positive	145	33.6%	32	35.6%		
Negative	287	66.4%	58	64.4%		
Living together with					5.923	0.025
Parents-in-law	157	36.3%	49	54.4%		
Parents	127	29.4%	10	11.1%		
Husband only	148	34.3%	31	34.5%		
Size of the house (m^2^)					8.487	0.054
< 60	47	10.8%	7	7.8%		
60 ~ 85	126	29.1%	48	53.3%		
85 ~ 120	175	40.5%	21	23.3%		
> 120	84	19.6%	14	15.6%		

See legend of Table 1. Data were from questionnaire results with 522 participants

### Exposure of some postnatal factors in women with and without significant PPD symptoms

As indicated in Table 3, the presence of some postnatal Chinese women-featured risk factors in participants with and without significant PPD symptoms was compared. Participants dissatisfied with husbands’ support, being looked after by parents-in-law, and with emotions vented on babies, were more likely to have significant PPD symptoms (*p* = 0.016, 0.024, 0.005, respectively). Moreover, those with cesarean delivery and mixed feeding also demonstrated an increased risk of developing significant symptoms of PPD (*p* = 0.029 and 0.013, respectively). The percentages of baby boy in the women with and without significant PPD symptoms were 47.8 and 58.8%, respectively, seemingly in accordance with the relevance of baby boy to prevention against PPD symptoms. However, no statistical difference between the two groups was observed (*p* = 0.132).

**Table 3:** Postnatal exposure of some social and clinical factors in women with and without significant PPD symptoms

	PPD^−^ women (n, %)		PPD^+^ women (n, %)		*χ*^2^	*p*
Self-reported husband’s support					7.965	0.016
Satisfied	397	91.9%	72	80.0%		
Dissatisfied	35	8.1%	18	20.0%		
Caregiver					6.329	0.024
Baby-sitter	68	15.7%	12	13.3%		
Parents-in-law	195	45.1%	53	58.9%		
Parents	113	26.2%	17	18.8%		
Parents from both sides	56	13.0%	8	9.0%		
Impulse of venting emotion to the newborn					7.135	0.005
Yes	86	19.9%	41	45.6%		
No	346	80.1%	49	54.4%		
Mode of delivery					4.754	0.029
Eutocia	258	59.7%	47	52.2%		
Cesarean	174	40.3%	43	47.8%		
Feeding pattern					3.389	0.013
Breast feeding	298	68.9%	21	23.3%		
Formula milk	25	5.9%	40	44.4%		
Mixed feeding	109	25.2%	29	32.3%		
Gender of the baby					2.763	0.132
Boy	254	58.8%	43	47.8%		
Girl	178	41.2%	47	52.2%		

Data were calculated from a total of 522 participants, who received telephone calls made during the 2nd to 4th postpartum months by three experienced investigators and answered all questions

### Association of some risk factors with significant PPD symptoms as identified by multiple logistic regression analysis

The adjusted odds ratios for factors that were significantly associated with greater PPD symptoms (as indicated in Tables 2 and 3) are summarized in Table 4. Postpartum women whose parents-in-law discriminate against a female baby (OR = 1.026, 95% CI 0.967, 1.087; *p* = 0.039), and who were dissatisfied with husband’s support (OR = 1.025, 95% CI 0.931, 1.128; *p* = 0.038) were at increased risk of developing significant PPD symptoms. Moreover, participants giving mixed feeding (OR = 1.002, 95% CI 1.000, 1.004; *p* = 0.017) and cesarean delivery (OR = 1.050, 95% CI 1.029, 1.071; *p* = 0.001) were also associated with the occurrence of significant PPD symptoms. However, the parents-in-law living together with a participant or taking care of her was not statistically associated with significant PPD symptoms.

**Table 4:** Multiple logistic regression analysis for the association of relevant risk factors with significant PPD symptoms

Characteristics	B	SE	Wald	*p*	OR	95% CI
Discrimination against a female baby by parents-in-law	0.141	0.053	6.543	0.039	1.026	0.967, 1.087
Dissatisfaction with husband’s support	0.742	0.359	4.286	0.038	1.025	0.931, 1.128
Mixed feeding	0.002	0.001	5.719	0.017	1.002	1.000, 1.004
Cesarean delivery	0.049	0.302	4.604	0.001	1.050	1.029, 1.071
Living with parents-in-law	0.388	0.293	2.408	0.199	1.474	1.058, 3.453
Parents-in-law taking care	0.648	0.035	1.844	0.121	1.048	1.000, 1.120

The list of characteristics was selected from those in Tables 2 and 3, based on the presence of statistical significance between women with and without significant PPD symptoms

## Discussion

The results of this study suggest that several Chinese population-featured factors, such as dissatisfaction with husband’s support, discrimination against a female baby by parents-in-law, mixed feeding and cesarean delivery, are associated with significant PPD symptoms. The presence of these factors means lack of social support, especially emotional support from the family.

The postpartum period (puerperium) is critical for the health of both the mother and infant, as relevant health promotion practice varies widely among individual families, which may adhere to different cultures/customs. In particular, modern health education about postpartum health is not commonly practiced by obstetrical doctors to the women in a hospital, due to the short service time in an outpatient visit (averaged 17.8 min as investigated from a hospital in China [13]), and doctors are usually too busy to provide personalized health education to hospitalized postpartum women. Therefore, the traditional Chinese regulation regarding postpartum health care is still followed by many families. The regulation requests that in 40 days after delivery, postpartum mothers should avoid the consumption of cold dishes/drinks, taking baths, and physical/outdoor activities, but should take sufficient nutritious animal foods, which is called “doing the month” as a critical period for postpartum women’s health [14, 15]. Even for mothers who have received higher education, violation of the rules of “doing the month” is seldomly understood or respected by their family members. In old times when industrialization had not been initiated, these rules had more positive than negative impacts on postpartum women, e.g., “doing the month” turned out to be effective for the prevention against uterine prolapse, since most women did a lot of physical (manual) labor with limited provision of foods, and no contraceptive tools existed to help them control reproduction, thus they usually delivered babies many times in their child-bearing years [16]. Nevertheless, in modern times these traditional postpartum rules may make the women feel like being imprisoned, especially in urban areas where life without daily communication with the social connections means a great suffering. In the meantime, postpartum women are susceptible to a series of symptoms, such as backache, breast problems, hemorrhoids, and anal fissure [17]. These symptoms may also disturb a woman’s mood; therefore, husbands may play an important role in caring, comforting and supporting their wives’ physical and mental health. In particular, in case that the parents-in-law (primarily, the mother-in-law) takes care of a postpartum woman, she has to deal with both her parents-in-law and her husband; in such ircumstances the husband has to maintain a balance between giving thanks to his parents and comforting his wife, and sometimes this balance is difficult to maintain, thus there might be adverse stimulants to the wife. Our results are consistent with a report from Shenzhen, Guangdong Province (in close proximity to Guangzhou), which suggested that living with parents-in-law may be a risk factor for PPD [18]. A husband’s words/attitude towards a postpartum wife, and his housework performance may have a substantial impact on his postpartum wife’s moods. Reports on women living in the mainland China [19] and Hongkong [20] suggest that family-related social support, dominated by marital relationship, followed by the relationship with mother-in-law, is the most important factor determining the rate of perinatal PPD symptoms. Our finding of the relevance of women’ dissatisfaction with husbands’ support to significant PPD symptoms is consistent with the previous reports.

In East Asia, the traditional “son preference” concept and related behavior are still dominant, at least in some areas, although many governments have made efforts to promote gender equality [21]. In a traditional Chinese society, people (especially elderly) regard boys, rather than girls, as their descendants. This old concept originated from ancient times when life depended primarily on physical labor, of which boys were considered to be more capable. Under this influence, some families desire a baby boy upon pregnancy/delivery; especially in case that the first child is a girl, the family more strongly desire that the second child be a boy [22], otherwise the family will have no chance to have a boy according to the current “two children policy” in China. Many elderly citizens do not understand that the gender of a baby is determined by a random match of germ cells from both sides: the mother and the father, and they may direct blame towards the postpartum mother, bringing her stress and worries. A mother generally and usually accepts the status of her baby, whether male or female, but pre-delivery judgement of a fetus by ultrasonic examination, be it male or female is legally forbidden in China (to prevent against selective induced abortion). Inferentially therefore, parents-in-law’s discrimination against a female baby means a decrease in social and moral support to a postpartum woman, which may contribute to the development of significant PPD symptoms.

Our study indicates that the postpartum mothers following cesarean delivery are associated with significant PPD symptoms. According to a report by the WHO in 2010, the rate of cesarean delivery in China reached 46.2% [23], which was the highest level in the world. Some factors prevalent in Chinese people may contribute to this situation. Due to the implementation of the “one-child policy” for more than 30 years (until 2016, when the Chinese government started to replace it with the “two-children policy”, considering the progressively decreasing birth rate) in China, and the request of females employed by government-owned institutions and industries for a late marriage (at ≥23 years) and a late pregnancy (at ≥25 years) (still active up to now), pregnancy at an advanced (≥ 35 years) age is relatively common. This may contribute to an elevated rate of difficult labor [24]. Moreover, due to the traditional Chinese culture-based concept, i.e., “the more food intake by a pregnant woman, the more healthy will be her baby”, and “a sedentary lifestyle is always preventive of miscarriage during pregnancy”, the Chinese pregnant women may be susceptible to gestational diabetes mellitus and other abnormalities in pregnancy, as compared to pregnant women in other countries [25, 26]. These pregnancy complications may contribute to a cesarean, rather than a vaginal, delivery. Cesarean delivery is a major disadvantage for the mother, and it may introduce some postoperative complications, e.g., endometritis, infection of abdominal incision, and irregular menstruation. Therefore, postpartum mothers who have given birth by cesarean delivery may have more postnatal symptoms and/or ailments as compared to those who have vaginal delivery, which may predispose the former to developing significant PPD symptoms [27, 28].

Among the participants recruited in this study, the rate of each feeding pattern, i.e., breast, mixed, and formula feeding, was 61.1, 26.4, and 12.5%, respectively. Further analysis indicated that mixed feeding was associated with significant PPD symptoms. In China, the availability of breast feeding is determined by the amount of breast milk produced and the convenience of a mother to feed her baby. Due to the commonly encountered occupational competition and pressure, many postpartum women cannot produce sufficient amount of breast milk (especially in a megacity like Guangzhou, from which the participants of this study were sampled out), although formula is not an economic source of milk for them, unlike women living in rural areas and working as farmers who usually have sufficient amounts of breast milk [29, 30]. Since a maternity leave for 4 to 6 months is ensured by the law and strictly implemented in China, the participants in this study were not back to work at the time of postnatal investigation. Even if a professional woman stays home taking care of her baby, her worrying about being discriminated by employers (in particular private employers who have more freedom to discontinue employment and change salary level) cannot be avoided. The employment stability, worktime flexibility and income maintenance to postpartum women are part of social support; lack of these support is associated with insufficient production of breastmilk. Moreover, breastfeeding may not be compatible with a woman’s decision to go back to work as early as possible, or her desire to keep fit by having a reduced amount of food; these conditions may lead to the adoption of mixed feeding [29]. Additionally, formula can be well distinguished from breast milk by, and uncomfortable for, the baby; thus, the baby cries and refuses to have formula, and this may annoy the mother and predispose her to PPD symptoms. Alternative, women who experience PPD symptoms may be more likely to switch to mixed feeding for some reasons, such as for getting more sleep. The observed association of mixed feeding with PPD symptoms may not be explained in a simple manner, and further research is required for a clarification.

### Study limitations

In this study, the postnatal questionnaire was completed in a relatively wide period of time, i.e., during their 2nd to 4th postpartum months. This may have compromised the homogeneity of the data. Additionally, since this is a cross-sectional study, no causality of the factors investigated can be concluded. Finally, this study points directly to the risk factors associated with elevated PPD symptoms, while it cannot be interpreted for clinically confirmed PPD women.

## Conclusion

The results of this study suggest that several social and clinical factors, some of them being characteristic of the Chinese population, are associated with significant PPD symptoms. These factors include discrimination against a female baby by parents-in-law, postpartum women being dissatisfied by husbands’ support, cesarean delivery, and mixed feeding. It is important that these factors are confirmed by continued studies, particularly with clinically confirmed PPD women in longitudinal investigations, so as to provide clues for the prevention against PPD, particularly in China.

## Data Availability

We would like to share the database upon individual request.

## Abbreviations

ANOVA: Analysis of Variance
BMI: Body Mass Index
OR: Odds Ratio
PPD: Postpartum Depression

## References

[1] Postpartum emotional disorders (2007). BC Reproductive Mental Health Program.

[2] Gavin NI, Gaynes BN, Lohr KN, Meltzer-Brody S, Gartlehner G, Swinson T (2005). Perinatal depression: a systematic review of prevalence and incidence. Obstet Gynecol.

[3] Gaynes BN, Gavin N, Meltzer-Brody S, Lohr KN, Swinson T, Cartlehner G, Brody S, Miller WC (2005). Perinatal depression: prevalence, screening accuracy, and screening outcomes. Evid Rep Technol Assess (Summ).

[4] Gao LL, Chan SW, Mao Q (2009). Depression, perceived stress, and social support among first-time Chinese mothers and fathers in the postpartum period. Res Nurs Health.

[5] Goodman SH, Brand SR, Hersen M, Gross AM (2008). Parental psychopathology and its relation to child psychopathology. Handbook of clinical psychology: Children and adolescents.

[6] Nestler EJ, Barrot M, DiLeone RJ, Eisch AJ, Gold SJ, Monteggia LM (2002). Neurobiology of depression. Neuron.

[7] Craddock N, Forty L (2006). Genetics of affective (mood) disorders. Eur J Hum Genet.

[8] Lee DT, Chung TK (2007). Postnatal depression: an update. Best Pract Res Clin Obstet Gynaecol.

[9] Garabedian MJ, Lain KY, Hansen WF, Garcia LS, Williams CM, Crofford LJ (2011). Violence against women and postpartum depression. J Women's Health (Larchmt).

[10] Segre LS, O’Hara MW, Arndt S, Stuart S (2007). The prevalence of postpartum depression: the relative significance of three social status indices. Soc Psychiatry Psychiatr Epidemiol.

[11] Cox J (1987). Origins and development of the 10 item Edinburgh depression scale. Perinatal psychiatry: use and misuse of the Edinburgh postnatal depression scale.

[12] Wang Y, Guo X, Lau Y, Chan KS, Yin L, Chen J (2009). Psychometric evaluation of the mainland Chinese version of the Edinburgh postnatal depression scale. Int J Nurs Stud.

[13] Xie Z, Or C (2017). Associations between waiting times, service times, and patient satisfaction in an endocrinology outpatient department: a time study and questionnaire survey. Inquiry.

[14] Liu YQ, Petrini M, Maloni JA (2015). “Doing the month”: postpartum practices in Chinese women. Nurs Health Sci.

[15] Raven JH, Chen Q, Tolhurst RJ, Garner P (2007). Traditional beliefs and practices in the postpartum period in Fujian Province, China: a qualitative study. BMC Pregnancy Childbirth.

[16] Lin YW, Chen CH (1999). Traditional health behaviors of postpartum women. Formosan J Med.

[17] Mao L, Ma L, Liu N, Chen B, Lu Q, Ying C, Sun X (2016). Self-reported health problems related to traditional dietary practices in postpartum women from urban, suburban and rural areas of Hubei province, China: the ‘zuò yuèzi’. Asia Pac J Clin Nutr.

[18] Wang YY, Li H, Wang YJ, Wang H, Zhang YR, Gong L, Ma J, Wang Y, Wang MZ, Qiu SX, Yuan SX (2017). Living with parents or parents-in-law and postpartum depression: a preliminary investigations in China. J Affect Disord.

[19] Siu BWM, Leung SSL, Ip P, Hung SF, O’Hara MW (2012). Antenatal risk factors for postnatal depression: a prospective study of Chinese women at maternal and child health centres. BMC Psychiatry.

[20] Lau Y, Fu D, Wong K (2008). The role of social support in helping Chinese women with perinatal depressive symptoms cope with family conflict. J Obstet Gynecol Neonatal Nurs.

[21] Clark S (2000). Son preference and sex composition of children: evidence from India. Demography.

[22] Chen Y (2009). Research on the social phenomena of male-preference in Chao-Shan area. J Guangzhou Panyu Polytech.

[23] Lumbiganon P, Laopaiboon M, Gülmezoglu AM, Souza JP, Taneepanichskul S, Ruyan P, Attygalle DE, Shrestha N, Mori R, Nguyen DH, Hoang TB, Rathavy T, Chuyun K, Cheang K, Festin M, Udomprasertgul V, Germar MJ, Yanqiu G, Roy M, Carroli G, Ba-Thike K, Filatova E, Villar J, World Health Organization global survey on maternal and perinatal Health Research Group (2010). Method of delivery and pregnancy outcomes in Asia: the WHO global survey on maternal and perinatal health 2007-08. Lancet.

[24] Cheng HM, Pei JH, Han F (2007). To investigate the effect of older pregnancy on the pregnant women and infants. J Pract Med.

[25] Ma G, Kong L (2006). China nutrition and health survey report.

[26] Su W, Li M, Yang H, Li Z (2009). Physical activities among pregnant women with normal glucose metabolism. J Nurs Sci.

[27] Li HT, Luo S, Trasande L, Hellerstein S, Kang C, Li JX, Zhang Y, Liu JM, Blustein J (2017). Geographic variations and temporal trends in cesarean delivery rates in China, 2008-2014. JAMA.

[28] Li F, Huang M, Zeng Y, Fang X (2017). Influence factors of postpartum depression in high-risk pregnant women. J Nurs.

[29] Liu J, Shi Z, Spatz D, Loh R, Sun G, Grisso J (2013). Social and demographic determinants for breastfeeding in a rural, suburban and city area of south East China. Contemp Nurse.

[30] Huang L, Li M, Bing S, Sun X, Pang X, Zhou L, Zeng G (2013). Breastfeeding behavior among infants aged 6-24months and difference between urban and rural areas in Southwest China. Chin J Perinat Med.

